# Supplementary material for: The Effect of Randomly Providing Nutri-Score Information on Actual Purchases in Colombia
Source: Nutrients. 2019 Feb 26;11(3):491. doi: 10.3390/nu11030491 (PMC6472000; doi:10.3390/nu11030491)
Supplement: Supplementary file 1 [file nutrients-11-00491-s001.pdf]

Supplementary Materials

The following tables present results when we impute the missing values in GPA by the average value of the non-missing values in GPA and omit the dummy variable to control for those cases. Table S1 is the simile of Table 2, Table S2 is the simile of Table 3, and so forth.

Table S1. Unconditional effect of providing Nutri-Score Label information on total expenditure and expenditure by color. Intention to treat (ITT) estimates. .

	Total	Green	Light Green	Orange	Pink	Red	Non-Labeled
	(1)	(2)	(3)	(4)	(5)	(6)	(7)
Estimated Effect	0.171**<br>[0.077]	0.204***<br>[0.074]	0.060<br>[0.034]	-0.037<br>[0.034]	0.006<br>[0.036]	0.037<br>[0.045]	-0.099**<br>[0.045]
N	*484*	*484*	*484*	*484*	*484*	*484*	*484*

Notes: In this table we impute the missing values in GPA by the average value of the non-missing values in GPA and omit the dummy variable to control for those cases. See Table 2 for further description. Robust standard errors in parenthesis. ** $p < 0.05$ *** $p < 0.01$.

Table S2. ITT and LATE effects of providing Nutri-Score Label information on total expenditure and expenditure by color, using different estimates.

	Total	Green	Light Green	Orange	Pink	Red	Non-Labeled
	(1)	(2)	(3)	(4)	(5)	(6)	(7)
A. Intention to treat (ITT)	0.171**<br>[0.077]	0.204***<br>[0.074]	0.06<br>[0.034]	-0.037<br>[0.034]	0.006<br>[0.036]	0.037<br>[0.045]	-0.099**<br>[0.045]
N	*484*	*484*	*484*	*484*	*484*	*484*	*484*
B. Local average treatment effect (LATE)	0.277**<br>[0.121]	0.331***<br>[0.117]	0.098<br>[0.054]	-0.060<br>[0.053]	0.010<br>[0.057]	0.060<br>[0.071]	-0.161**<br>[0.070]
N	*484*	*484*	*484*	*484*	*484*	*484*	*484*

Notes: This table follows the same specification as Table B1. Robust standard errors in parenthesis. ** $p < 0.05$ *** $p < 0.01$.

Table S3. Effect of providing Nutri-Score Label information on expenditure by color, conditional on buying at least one item of a given color.

	Green	Light Green	Orange	Pink	Red	Non-Labeled
	(1)	(2)	(3)	(4)	(5)	(6)
Estimated Effect	0.341**<br>[0.143]	0.673<br>[0.320]	-1.703<br>[0.923]	-0.007<br>[0.115]	0.046<br>[0.047]	-0.047<br>[0.050]
N	*137*	*33*	*32*	*83*	*223*	*179*

Notes: The dependent variable in this table is the amount of money spent by each person (or on each purchase) on items of the indicated color in each column, conditional on buying at least one item of the same color. This table uses the same covariates as in Table B1. Robust standard errors in parenthesis. ** $p < 0.05$ *** $p < 0.01$.

Table S4. ITT and LATE effects of providing Nutri-Score Label information on the probability of purchasing at least one item of a particular color using a linear probability model (LPM).

	**Green**	**Light Green**	**Orange**	**Pink**	**Red**	**Non-Labeled**
	(1)	(2)	(3)	(4)	(5)	(6)
A. Intention to Treat (ITT)	0.099**<br>[0.042]	0.026<br>[0.023]	-0.011<br>[0.024]	-0.002<br>[0.035]	0.016<br>[0.045]	-0.098**<br>[0.045]
N	*484*	*484*	*484*	*484*	*484*	*484*
B. Local average treatment effect (LATE)	0.161**<br>[0.066]	0.041<br>[0.036]	-0.018<br>[0.038]	-0.003<br>[0.055]	0.026<br>[0.071]	-0.159**<br>[0.071]
N	*484*	*484*	*484*	*484*	*484*	*484*

Notes: The dependent variable in this table is a dummy that takes the value of one if the person bought at least one item of the color indicated in each column, and zero otherwise. All other specifications are the same as in Table B1. Robust standard errors in parenthesis. ** $p < 0.05$ *** $p < 0.01$.

Table S5. ITT and LATE effects of providing Nutri-Score Label information over nutritional content of bought items in a standardized serving size (100 g for solid or 100 mL for liquid), by nutrient.

	**Proteins (g)**	**Calories (kcal)**	**Sugars (g)**	**Sodium (mg)**	**Saturated Fats (g)**	**Fibers (g)**
	(1)	(2)	(3)	(4)	(5)	(6)
A. Intention to Treat (ITT)	2.812***<br>[0.856]	66.672<br>[111.434]	-2.677<br>[2.505]	38.074<br>[25.123]	0.031<br>[1.218]	0.654<br>[0.578]
N	*393*	*393*	*393*	*393*	*393*	*393*
C. Local average treatment effect (LATE)	4.421***<br>[1.319]	104.826<br>[169.372]	-4.210<br>[3.834]	59.863<br>[38.181]	0.049<br>[1.854]	1.028<br>[0.882]
N	*393*	*393*	*393*	*393*	*393*	*393*

Notes: Robust standard errors in parenthesis. ** $p < 0.05$ *** $p < 0.01$.
